# Earnings and work loss from 5 years before to 5 years after bariatric surgery: A cohort study

**DOI:** 10.1371/journal.pone.0285379

**Published:** 2023-05-18

**Authors:** Mattias Norrbäck, Martin Neovius, Johan Ottosson, Ingmar Näslund, Gustaf Bruze

**Affiliations:** 1 Department of Medicine, Clinical Epidemiology Division, Karolinska Institutet, Stockholm, Sweden; 2 Faculty of Medicine and Health, Department of Surgery, Örebro University, Örebro, Sweden; Taipei Medical University, TAIWAN

## Abstract

**Background:**

The personal economic impact of bariatric surgery is not well-described.

**Objectives:**

To examine earnings and work loss from 5 years before to 5 years after bariatric surgery compared with the general population.

**Setting:**

Nationwide matched cohort study in the Swedish health care system.

**Methods:**

Patients undergoing primary bariatric surgery (n = 15,828) and an equal number of comparators from the Swedish general population were identified and matched on age, sex, place of residence, and educational level. Annual taxable earnings (primary outcome) and annual work loss (secondary outcome combining months with sick leave and disability pension) were retrieved from Statistics Sweden. Participants were included in the analysis until the year of study end, emigration or death.

**Results:**

From 5 years before to 5 years after bariatric surgery, earnings increased for patients overall and in subgroups defined by education level and sex, while work loss remained relatively constant. Bariatric patients and matched comparators from the general population increased their earnings in a near parallel fashion, from 5 years before (mean difference -$3,489 [95%CI -3,918 to -3,060]) to 5 years after surgery (-$4,164 [-4,709 to -3,619]). Work loss was relatively stable within both groups but with large absolute differences both at 5 years before (1.09 months, [95%CI 1.01 to 1.17]) and 5 years after surgery (1.25 months, [1.11 to 1.40]).

**Conclusions:**

Five years after treatment, bariatric surgery had not reduced the gap in earnings and work loss between surgery patients and matched comparators from the general population.

## Introduction

Over 120 million people worldwide live with obesity (Body Mass Index [BMI]≥30 kg/m^2^) [1], associated with a higher risk of poor health, and an increased risk of premature death [2]. Men and women with obesity are also less likely to be employed, have higher levels of sick leave, have more disability-related income [3], and receive lower earnings [4,5].

Bariatric surgery is the only known treatment for obesity that results in large and durable weight loss and improved control or remission of type-2 diabetes [6,7]. Despite the well-documented positive impact of bariatric surgery on physical health, its personal economic impact has been studied much less. The relationship between earnings and bariatric surgery remains unclear, particularly when stratifying by patient sex and level of education.

The available evidence has been inconclusive and mostly limited to studies with small samples, short follow-up, self-reported outcome measures, and lack of comparators [8–10]. We recently reported results from an analysis of female bariatric surgery patients of fertile age and BMI-matched comparators suggesting that the pre-operative difference in earnings between these two groups remains for as long as five years after surgery [11]. Similar results for sick leave and disability pension (work loss) were likewise found in a study comparing bariatric surgery patients to age-sex-income matched comparators from the general population [12]. In summary, recent findings suggest that bariatric surgery may have limited impact on earnings and work loss for women, but this relationship has, to our knowledge, not been confirmed in men.

The aim of this study was to examine changes in earnings (primary outcome) and work loss (secondary outcome) measured objecively for bariatric surgery patients and subgroups categorized by educational level and sex, from 5 years before to 5 years after surgery. Earnings and work loss were also compared between bariatric surgery patients and general population comparators matched on age, sex, place of residence, and educational level.

## Methods

This nationwide matched cohort study was conducted using patients identified in the Scandinavian Obesity Surgery Registry (SOReg) [13,14] and matched comparators from the Swedish general population retrieved from the Swedish Total Population Register [15].

Data on outcomes and socio-economic and health-related factors were obtained from nationwide registers in Sweden and linked to patients and matched comparators using the unique personal identification number of each Swedish resident [16]. Ethical approval was granted by the regional ethics committee in Stockholm (No. 2013/730-31).

SOReg [13,14] is a nationwide, prospective register for bariatric surgery started in 2007. It has been estimated to cover 98.5% of all bariatric procedures in Sweden. Data are stored electronically and recorded as part of clinical practice.

### Study cohorts

#### Bariatric surgery cohort

We included patients from SOReg who underwent primary bariatric surgery during 2007–2014 and who were between ages 30 and 55 years at the time of surgery. The purpose of this age restriction was to exclude students and individuals early in their work career (<30y), and individuals potentially retiring during follow-up (>55y). We also wanted patients to have sufficient time to be able to establish or reestablish themselves professionally after their surgery, before attaining the age of retirement (or preterm retirement). To account for potential changes over time in patient characteristics and labor market conditions, patients were divided by year of surgery (2007–2008, 2009–2010, 2011–2012, and 2013–2014). Patients were also divided into subgroups based on educational level and sex.

#### Matched cohort

We also matched bariatric surgery patients (1:1) who had primary surgery between 2007 and 2011 to comparators from the Swedish general population on age, sex, place of residence (municipality), and educational level (primary school, high school, university).

### Main outcome measures and follow-up

Earnings data (primary outcome) were retrieved from 2002 to 2016 and work loss data (secondary outcome) from 2002 to 2014. These outcomes are recorded in the Swedish Longitudinal Integration Database for Health Insurance and Labor Market Studies (LISA) that covers the adult population of Sweden [17].

Our earnings data originate from reports made by employers to the Swedish Tax Agency listing the total taxable earned gross income of each employee in a calendar year. The earnings data were adjusted for annual inflation using the Swedish consumer price index and converted to 2016 US dollars using the exchange rate from July 1^st^ 2016 (1 USD = 8.43 SEK). Annual taxable earnings retrieved from government records are an objective and reliable measure of a change in gainful employment. Not only can earnings measure income effects through a job change within or between companies, but they also capture shifts from permament to temporary positions or from full-time to part-time positions. Temporary and part-time positions are more common among people with chronic conditions such as obesity and type-2 diabetes.

The Swedish welfare system provides compensation for sick leave and disability pension, which may be complete or partial. Sick leave is reimbursed by the employer from day 2 to day 14. Episodes longer than 14 days are recorded by the Social Insurance Agency, which reimburses the employee due to lost income from day 15 and onwards. An employee with a 25% reduced work ability or more (as evaluated by a physician) that is expected to last for at least 1 year, may receive disability pension (on a time-limited or permanent basis). For an employee with disability pension, work ability is re-evaluated regularly to improve rehabilitation back to work.

Our measure of work loss is the sum of sick leave and disability pension. Combining these two types of benefits gives a unified measure of work loss that is both comparable over time and insensitive to institutional changes that may move individuals between these two benefit systems. Complete data on work loss from LISA were available for all participants, regardless of the extent (full time/part time) or type of work.

The bariatric surgery cohort was divided by surgery year so that patients who had surgery 2007–2008 were followed the longest, and patients who had surgery 2013–2014 were followed the shortest. In total, all bariatric patients were followed from 5 years before to a maximum of 7 years after the surgery year. Subgroups of the bariatric surgery cohort based on educational level and sex were followed from 5 years before to 5 years after surgery. The matched cohort of bariatric surgery patients and comparators from the general population were followed from 5 years before to 5 years after the surgery/index year.

Participants who died (patients: 2.6%; comparators: 1.5%) or emigrated (patients: 1.0%; comparators: 1.6%) were followed until the year before death or emigration.

### Covariates

Educational level and marital status (married/not married) were retrieved from LISA. Annual number of hospital days and annual number of outpatient hospital visits were retrieved from the Swedish National Patient Register. Obesity-related comorbidities measured prior to the index date including antidiabetic drug use, antihypertensive therapy, psychotropic drug use, and lipid modifying therapy were retrieved from the Swedish Prescribed Drug Register (Anatomical Therapeutical Category [ATC] codes in S1 Table in S1 Appendix). Further, any circulatory disease and psychiatric disease (outpatient and inpatient hospital care) were retrieved from the Swedish National Patient Register (International Classification of Diseases [ICD] codes in S1 Table in S1 Appendix).

### Statistical analysis

#### Bariatric surgery cohort

For the bariatric surgery cohort without comparators, earnings and work loss were first examined in 4 bariatric subgroups by year of surgery (2007–2008, 2009–2010, 2011–2012, and 2013–2014). The bariatric surgery cohort was also divided by educational level (primary school, high school, and university) and sex. Results are presented graphically as annual mean taxable earnings and annual mean work loss.

#### Matched cohort

In the matched cohort, we compared the earnings and work loss of bariatric surgery patients with the matched general population comparators. Results are presented as annual mean taxable earnings and work loss for both groups (with 95% confidence intervals in S3 and S4 Tables in S1 Appendix), and annual mean differences of earnings and work loss between the groups with 95% confidence intervals (general population comparators were used as reference group). The difference between surgery patients and their matched comparators was adjusted (through matching) for age, sex, place of residence, and education. As a sensitivity analysis, we also computed the adjusted mean difference between surgery patients and general population comparators using linear regression. In this sensitivity analysis, the difference between surgery patients and their matched comparators was adjusted (through matching) for age, sex, place of residence, and education, and further adjusted (through linear regression) for marital status, annual number of hospital days, and annual outpatient hospital visits in the year of observation.

In a separate analysis, the matched cohort was divided into patient-comparator subgroups by educational level (primary school, high school, and university), earnings before surgery (quartiles), and work loss before surgery (quartiles). Statistical analyses were carried out using Stata version 16.2 and SAS version 9.4.

## Results

### Descriptive characteristics

From 2007–2014, 31,791 patients aged 30-55y underwent bariatric surgery (S1 Fig in S1 Appendix), with the two most common procedures being gastric bypass (94%) and sleeve gastrectomy (4%). The mean age was 42 years, 76% were women, mean pre-surgery BMI was 42kg/m^2^, and 23% had a university education. The year before surgery, patients had annual mean earnings of 24,100 USD with a mean work loss of 2.0 months per year (S2 Table in S1 Appendix).

In the matched cohort, 15,828 bariatric surgery patients who had surgery between 2007 and 2011 and 15,828 general population comparators were included (Table 1). Prior to the index date, bariatric surgery patients were more often in contact with health-care and were more likely to have obesity-related comorbidities than general population comparators (Table 1).

**Table 1:** Characteristics of bariatric surgery patients and matched comparators from the general population.

	Matched cohort†	
	Bariatric surgery patients	General population comparators
N	15,828	15,828
Women, n (%)	11,987 (75.7)	11,987 (75.7)
Age (years)	42.4 (6.8)	42.4 (6.8)
**Educational level, n (%)**		
Primary school	1724 (10.9)	1724 (10.9)
High school	10,394 (65.7)	10,394 (65.7)
University	3701 (23.4)	3701 (23.4)
*Education missing*	*9 (0*.*1)*	*9 (0*.*1)*
Earnings (U.S Dollars $*)		
Mean (SD)	23,600 (21,000)	27,300 (22,200)
25^th^ percentile	3100	9600
50^th^ percentile (median)	24,800	28,700
75^th^ percentile	35,700	38,100
**Work loss months, Mean (SD)**	2.3 (4.2)	1.1 (3.1)
Sick leave	0.8 (2.4)	0.3 (1.5)
Disability pension	1.4 (3.6)	0.7 (2.7)
Married, yes	7302 (46.1)	7413 (46.8)
**Health-care use, Mean (SD)** **		
Number of outpatient visits	2.31 (3.38)	1.07 (2.78)
Number of hospital days	0.80 (6.96)	0.58 (6.40)
**History of comorbidities, n (%)**		
Antidiabetic Drug Use	2636 (16.7)	382 (2.4)
Antihypertensive Therapy	4702 (29.7)	1371 (8.7)
Circulatory Disease	4153 (26.2)	1685 (10.6)
Psychiatric Disease	3512 (22.2)	1984 (12.5)
Psychotropic Drug Use	7561 (47.8)	4241 (26.8)
Lipid Lowering Therapy	2386 (15.1)	575 (3.6)

SOReg = Patients from the Scandinavian Obesity Surgery Registry.

^†^Population comparators are matched to SOReg patients on age, sex, place of residence (municipality level), and educational level (primary school, high school, and university).

* Collected the year before surgery or index year. Currency units presented as 2016 U.S. dollars (converted from Swedish crowns, based on exchange rate July 1st, 2016, adjusted for Swedish annual inflation according to Swedish CPI).

** Health-care use during the last five years before surgery.

### Earnings and work loss for the bariatric surgery cohort

#### Overall

**Fig 1:**
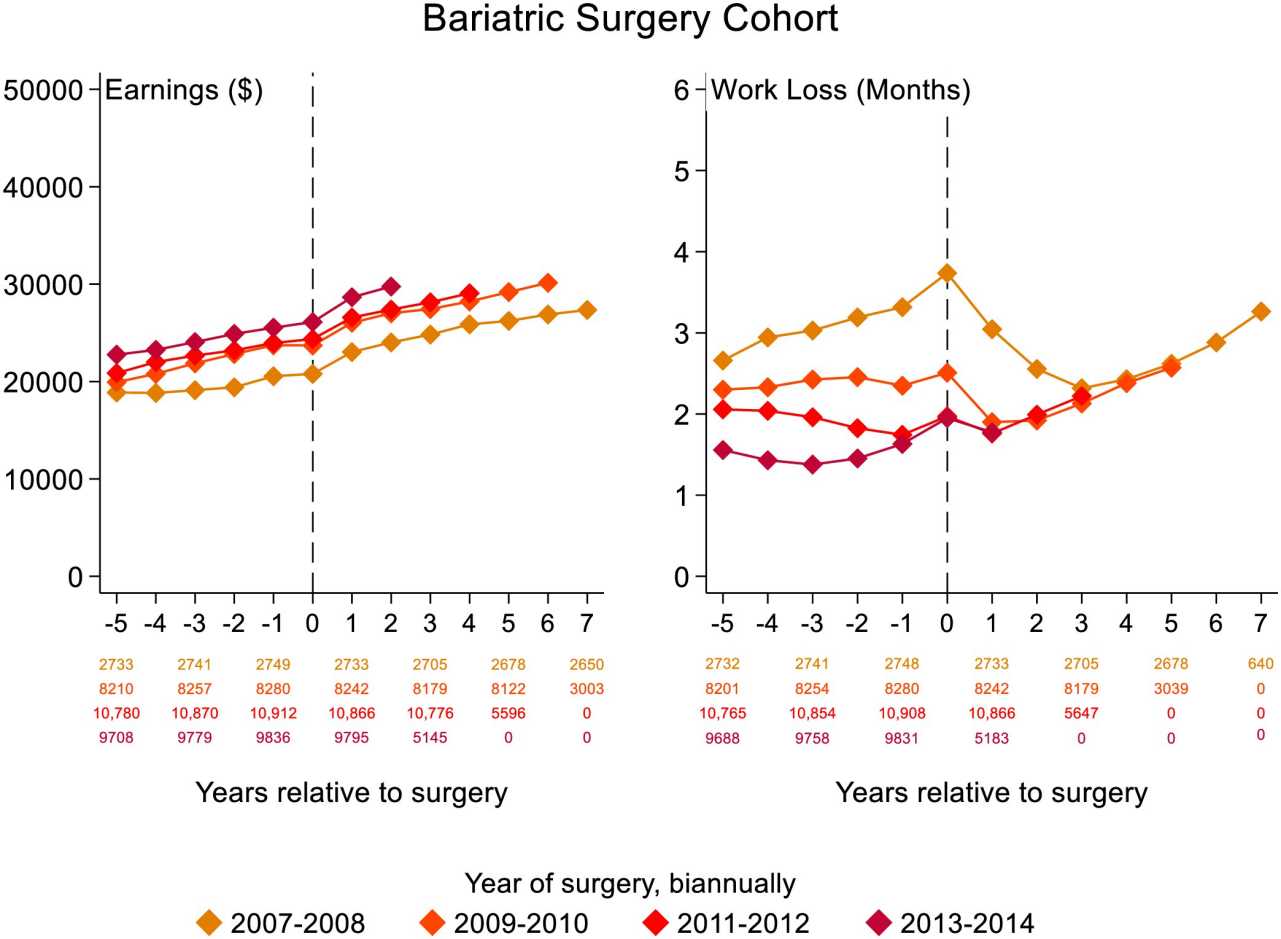
Development of annual earnings in 2016 U.S. dollars and work loss for bariatric surgery patients. Vertical dashed line is surgery year.

Bariatric surgery patients increased their earnings in a near-linear fashion, except for a small decrease or leveling off during the year of surgery, which was mirrored by an increase in work loss (Fig 1). Work loss was at similar levels 5 years after versus 5 years before surgery and peaked in the year of surgery, with findings consistent when stratified by year of surgery (Fig 1).

#### Subgroups

**Fig 2:**
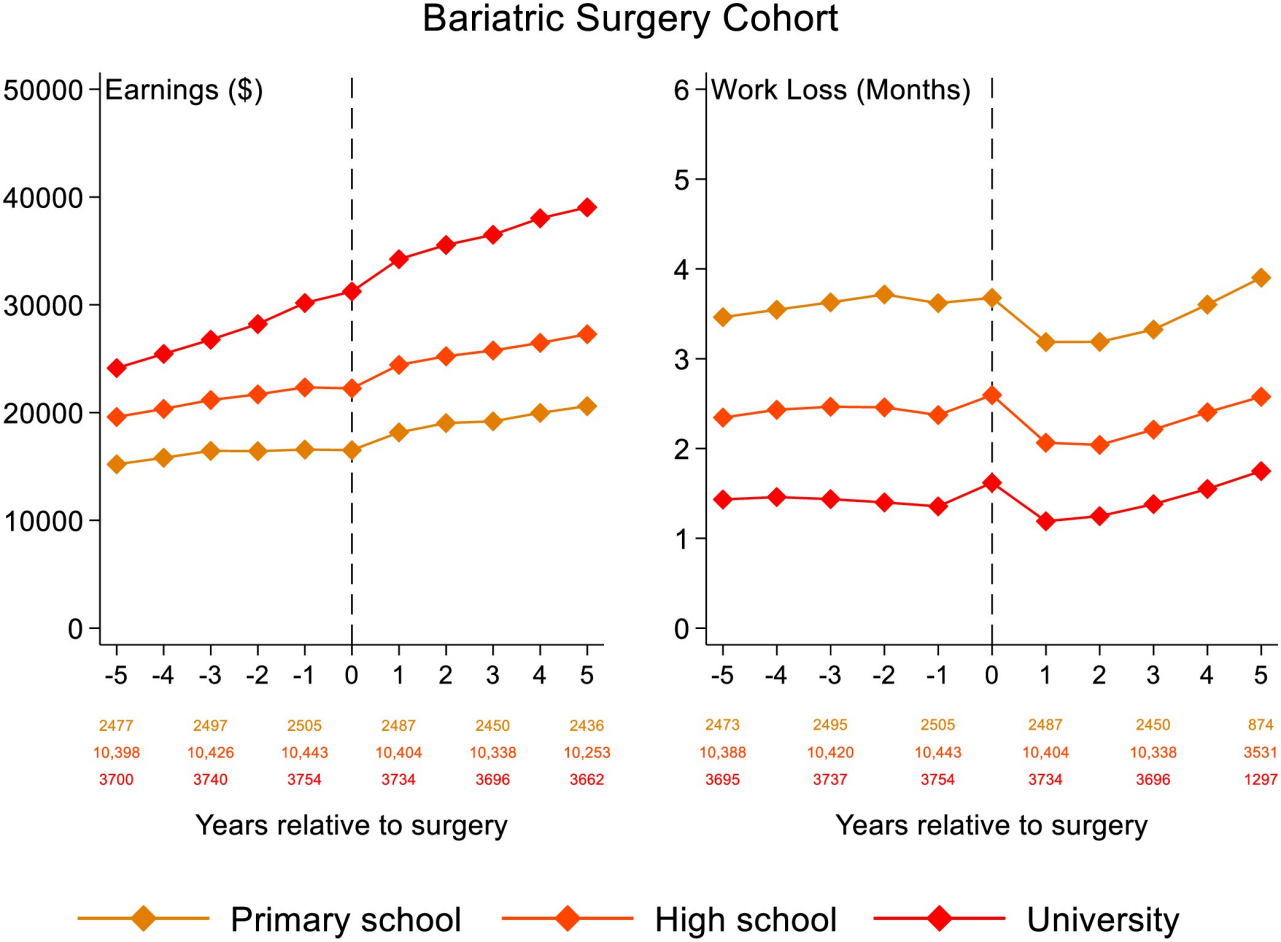
Development of annual earnings in 2016 U.S. dollars and work loss for the bariatric surgery cohort, by educational level. Vertical dashed line is surgery year.

For the bariatric cohort subgroups defined by educational level (Fig 2), earnings increased close to linearly from 5 years before to 5 years after surgery, but with earnings leveling off during the year of surgery. Patients with university education had the steepest earnings development. Earnings did not develop differently between men and women (S2 Fig in S1 Appendix), although men earned on average about $10,000 more annually than women.

Work loss was similar 5 years before surgery compared to 5 years after surgery for all education subgroups (Fig 2). During the year of surgery, work loss peaked for all subgroups. Patients with primary school education had the highest work loss (≈4 months per year). Women had greater annual work loss on average than men (S2 Fig in S1 Appendix).

### Earnings and work loss in the matched cohort

#### Overall

**Fig 3:**
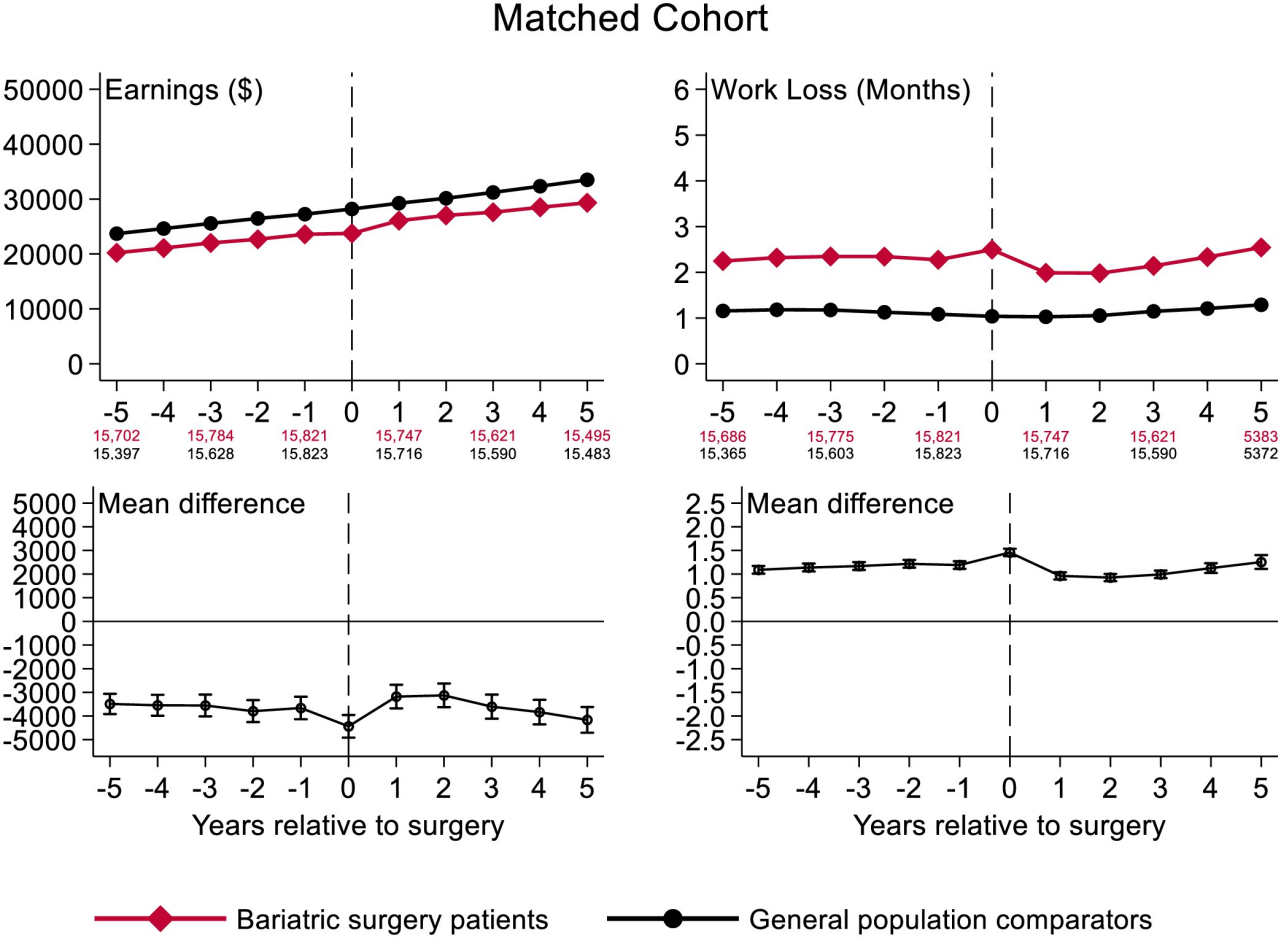
Development of annual earnings in 2016 U.S. dollars and work loss for the bariatric surgery patients and comparators from the general population matched on age, sex, place of residence, and educational level (primary school, high school, or university training). Calculated mean differences are earnings (or work loss) for bariatric surgery patients minus earnings (or work loss) for comparators from the general population. Vertical dashed line is surgery year for patients and index year for general population comparators.

Earnings developed similarly for bariatric surgery patients and matched general population comparators from 5 years before to 5 years after surgery (Fig 3), although the comparators earned more on average both 5 years before (mean difference -$3,489 [95%CI -3,918 to -3,060]) and 5 years after surgery (-$4,164 [-4,709 to -3,619]). Similar trends were observed when further adjusting for marital status, annual number of hospital days, and annual outpatient hospital visits in a sensitivity analysis, although the additional adjustment reduced differences between bariatric patients and comparators 5 years before (adjusted mean difference -$2,905 [95%CI -3,333 to -2,478)] and 5 years after surgery [-$2,975 (-3,515 to -2,436; S3 Fig in S1 Appendix]. When winsorizing annual taxable earnings at the 1^st^ and 99^th^ percentile, overall trends in earnings were similar as in the main analysis with a mean difference of -$3,325 [-3,706 to -2,945] 5 years before surgery and a mean difference of -$4,069 [-4,557 to -3,581] 5 years after surgery (S4 Fig in S1 Appendix).

Work loss also developed similarly for bariatric surgery patients and matched general population comparators from 5 years before to 5 years after surgery (Fig 3). The average annual difference in work loss 5 years before surgery (1.09 months, [95%CI 1.01 to 1.17]) was similar to the annual difference 5 years after surgery (1.25 months, [95%CI 1.11 to 1.40]). During the surgery/index year, earnings dropped and work loss peaked for bariatric surgery patients compared with matched general population comparators (S3 Fig in S1 Appendix), but this difference was attenuated in the sensitivity analysis.

#### Subgroups

Earnings increased over time for bariatric surgery patients and matched general population comparators in all education subgroups (S5 Fig in S1 Appendix). Larger annual differences in earnings between bariatric patients and matched comparators were found in the subgroup with primary school education relative to the subgroup with university education.

Annual work loss was similar over time between patients and comparators in all education subgroups (S5 Fig in S1 Appendix). In the subgroup with primary school education, patients had approximately 3.5 months of work loss per year compared to 2.5 months for comparators.

Patients with the lowest earnings before surgery (1^st^ quartile) had the lowest earnings after surgery (S6 Fig in S1 Appendix). Similarly, patients with the highest work loss before surgery (4^th^ quartile) had the largest decrease in work loss after surgery (~2 months reduction) but still with the highest absolute levels of work loss (S7 Fig in S1 Appendix).

## Discussion

The results did not indicate any significant increase in annual earnings and reduction in work loss after treatment for bariatric surgery patients compared with the matched general population. Both before and after surgery, absolute differences in annual earnings versus matched general population comparators remained similar at about $3,500–4,000 and differences in work loss remained similar at about 1 month. As in the general population, mean earnings and work loss increased with age in bariatric surgery patients.

The results regarding earnings in this study are consistent with findings from our previous study of women of fertile age suggesting that bariatric surgery had little impact on the five-year development of objectively measured earnings [11]. In our previous study, we compared earnings of female bariatric patients with their full sisters (partly accounting for familial and genetic factors) as well as comparators matched on various characteristics including BMI [11]. This study compliments our earlier work by including results on earnings for male patients, and female patients who did not have children. The lack of improvement in earnings after bariatric surgery is supported by two previous cohort studies from the U.S. [9] and Norway [18] as well as one study from the U.S [19]. These studies found no overall improvements in paid work over 3 to 5 years after bariatric surgery compared with before surgery. In contrast, three studies found evidence of a positive impact of bariatric surgery on earnings. A retrospective study from the UK, following 59 patients for 14 months on average, found a 32% increase in patients who reported having paid work after bariatric surgery [20]. Similar results were found in a Norwegian study following 51 patients for up to 48 months after bariatric surgery [21]. Last, the US study [19] followed 304 bariatric surgery patients and found a small increase in the number of working hours during the first year following surgery, compared with the year prior to surgery. However, the three studies indicating a positive effect of bariatric surgery did not include a comparator group and had different self-reported outcomes which taken together could explain the inconsistency in findings.

Concerning work loss, our findings overall and by educational level, are consistent with a previous Swedish study showing that bariatric patients had more sickness benefits before and up to 3 years after surgery, relative to age-sex-income matched comparators from the general population [12]. In contrast, evidence from the Swedish Obese Subjects (SOS) study of surgery (mostly using older bariatric procedures) versus controls receiving standard treatment [22,23] and one uncontrolled Norwegian study [18] suggests small reductions in sick leave [18,23] and a modest reduction in disability pension in young men [22]. Relative to previous Swedish studies on work loss, the current study adds longer follow-up for data on sick leave and disability pension from patients treated with more recent bariatric procedures.

We found that earnings and work loss did not differ over time for Swedish bariatric surgery patients relative to matched comparators from the general population. There are several potential explanations for these results.

First, bariatric surgery improves health and quality of life [24,25] which may reduce sick leave and disability pension [18,22,23]. However, this may not be the case for bariatric patients with high levels of work loss before surgery, who may be weakly attached to the labor market because of work disability or other health problems. This was partly confirmed in our supplementary analysis where people with high levels of work loss before surgery also had the highest levels of work loss after surgery, although there was an indication that work loss reduction was highest in this group. Further, evidence from two Nordic studies of chronically ill patients with rheumatoid arthritis found that high levels of work loss before diagnosis was the strongest predictor of future work loss after diagnosis [26,27].

Second, bariatric surgery patients have greater socio-economic disadvantages compared with the general population, both in the U.S. and Sweden [28–30]. For instance, low occupational status in combination with a stressful work environment [31] could negatively influence health status over time [32]. This deterioration may counteract any earnings gains after bariatric surgery. However, our findings were consistent in all education subgroups and were robust in our sensitivity analysis when adjusting for marital status, arguing against such an explanation in this context.

Third, bariatric patients are at a higher risk of being absent from work due to post-surgical recovery and complications leading to reduced earnings. In our sensitivity analysis, we took into account the annual number of hospital days and outpatient hospital visits. This adjustment reduced differences between patients and population comparators, especially during the year of surgery.

Fourth, bariatric surgery improves physical health over time, but the impact on mental health is not as well-understood. A meta-analysis of 11 RCTs, including over 700 patients, found no improvement in mental health-related quality of life after bariatric surgery compared to non-surgical interventions [33]. Other studies have suggested that bariatric surgery patients may be at an increased risk of substance abuse [34,35], self-harm [36], and suicide [37]. In our previous study using BMI-matched comparators, we partly accounted for psychiatric health. It is possible that post-surgical mental health problems manifest themselves differently in this cohort with female and male bariatric patients.

Fifth, people with obesity, especially women, face stigmatization [4]. The weight stigma negatively influences working life through strong cultural norms that tend to persist over long periods of time. Such weight stigma could limit the beneficial impact of health improvements that would otherwise raise the earnings of bariatric surgery patients (≥75% women), and could delay increases in earnings beyond our follow-up time.

Lastly, the mean age at treatment of patients and comparators in this study is above 40 years. At this age and during subsequent follow-up, overall job mobility and the tendency to change profession is lower than for younger workers, which could also reduce the impact of bariatric surgery on earnings.

Despite the reasons we mention above that may cancel out the positive effects of bariatric surgery on future earnings, it is possible to view the post-surgical earnings and work loss in our bariatric patients as better than expected, as there is no declining trend in this patient group.

### Strengths and limitations

This study has several strengths, including the use of objectively measured data on a large sample of bariatric patients, which provides reliable estimates of changes in earnings and work loss overall and across sex and education subgroups relative to the general population. The outcomes were measured from 5 years before to 5 years after surgery enabling examination of trend breaks, including breaks around the time of surgery. This can be a critical time period when patients are likely to be absent from work (with reduced earnings) due to post-surgical recovery.

One limitation of our study is that we did not have information on BMI in comparators and can only speculate about the possible effects we may have observed if we had been able to match comparators on BMI and psychiatric factors. We have previously reported similar findings to this study when comparing female bariatric surgery patients with a comparator group matched on BMI and psychiatric health, suggesting that these factors play a lesser role in our results.

Finally, it is important to note that our findings primarily relate to patients receiving gastric bypass surgery in a high-income country with high workforce participation and extensive welfare benefits. Therefore, the generalizability of our results to non-Nordic countries, particularly to the U.S., may be limited. However, our focus on changes over time rather than absolute levels of earnings reduce the sensitivity of our results to differences between labor markets across countries. Nevertheless, our results are primarily applicable to countries with similar labor market and social security institutions as Sweden.

### Conclusions

We could not observe any catch-up effect in earnings for the surgical patients relative to comparators from the general population. Five years after bariatric surgery, the patients continued to earn less in the Swedish labor market than the general population and to have more days with sick leave or disability pension from the Swedish welfare system.

## Supporting information

S1 Appendix Supplementary web appendix—Contains supporting figures and tables. (PDF)

## References

[1] Risk Factor Collaboration N. C. D.. Worldwide trends in body-mass index, underweight, overweight, and obesity from 1975 to 2016: a pooled analysis of 2416 population-based measurement studies in 128.9 million children, adolescents, and adults. Lancet. 2017;390(10113):2627–42. 29029897 10.1016/S0140-6736(17)32129-3 PMC5735219

[2] G. B. D. Obesity Collaborators, Afshin A, Forouzanfar MH, Reitsma MB, Sur P, Estep K, et al. Health Effects of Overweight and Obesity in 195 Countries over 25 Years. N Engl J Med. 2017;377(1):13–27. doi: 10.1056/NEJMoa1614362 28604169 PMC5477817

[3] Neovius K, Johansson K, Kark M, Neovius M. Obesity status and sick leave: a systematic review. Obes Rev. 2009;10(1):17–27. doi: 10.1111/j.1467-789X.2008.00521.x 18778315

[4] Puhl RM, Heuer CA. The stigma of obesity: a review and update. Obesity (Silver Spring). 2009;17(5):941–64. doi: 10.1038/oby.2008.636 19165161

[5] Cawley J. An economy of scales: A selective review of obesity’s economic causes, consequences, and solutions. J Health Econ. 2015;43:244–68. doi: 10.1016/j.jhealeco.2015.03.001 26279519

[6] Courcoulas AP, Gallagher JW, Neiberg RH, Eagleton EB, DeLany JP, Lang W, et al. Bariatric Surgery vs Lifestyle Intervention for Diabetes Treatment: 5-Year Outcomes From a Randomized Trial. J Clin Endocrinol Metab. 2020;105(3). doi: 10.1210/clinem/dgaa006 31917447 PMC7032894

[7] Sjostrom L, Peltonen M, Jacobson P, Ahlin S, Andersson-Assarsson J, Anveden A, et al. Association of bariatric surgery with long-term remission of type 2 diabetes and with microvascular and macrovascular complications. JAMA. 2014;311(22):2297–304. doi: 10.1001/jama.2014.5988 24915261

[8] Naslund II, Agren G. Social and Economic Effects of Bariatric Surgery. Obes Surg. 1991;1(2):137–40. doi: 10.1381/096089291765561132 10775905

[9] Alfonso-Cristancho R, King WC, Mitchell JE, Ramanathan R, Sullivan SD, Belle SH, et al. Longitudinal Evaluation of Work Status and Productivity After Bariatric Surgery. Jama-J Am Med Assoc. 2016;316(15):1595–7. doi: 10.1001/jama.2016.12040 27755626 PMC5314899

[10] Vayr F, Charras L, Savall F, Soulat JM, Ritz P, Herin F. The Impact of Bariatric Surgery on Employment: A Systematic Review. Bariatr Surg Pract P. 2018;13(2):54–63.

[11] Norrback M, Neovius M, Ottosson J, Naslund I, Bruze G. Earnings and employment for women after bariatric surgery: a matched cohort study. Int J Obes (Lond). 2021;45(4):766–75. doi: 10.1038/s41366-021-00737-1 33495524

[12] Jonsson E, Ornstein P, Goine H, Hedenbro JL. Diabetes Resolution and Work Absenteeism After Gastric Bypass: a 6-Year Study. Obes Surg. 2017;27(9):2246–52. doi: 10.1007/s11695-017-2642-5 28293901 PMC5562776

[13] Hedenbro JL, Naslund E, Boman L, Lundegardh G, Bylund A, Ekelund M, et al. Formation of the Scandinavian Obesity Surgery Registry, SOReg. Obes Surg. 2015;25(10):1893–900. doi: 10.1007/s11695-015-1619-5 25703826

[14] Sundbom M, Naslund I, Naslund E, Ottosson J. High acquisition rate and internal validity in the Scandinavian Obesity Surgery Registry. Surg Obes Relat Dis. 2020. doi: 10.1016/j.soard.2020.10.017 33243667

[15] Ludvigsson JF, Almqvist C, Bonamy AKE, Ljung R, Michaelsson K, Neovius M, et al. Registers of the Swedish total population and their use in medical research. Eur J Epidemiol. 2016;31(2):125–36. doi: 10.1007/s10654-016-0117-y 26769609

[16] Ludvigsson JF, Otterblad-Olausson P, Pettersson BU, Ekbom A. The Swedish personal identity number: possibilities and pitfalls in healthcare and medical research. Eur J Epidemiol. 2009;24(11):659–67. doi: 10.1007/s10654-009-9350-y 19504049 PMC2773709

[17] Ludvigsson JF, Svedberg P, Olen O, Bruze G, Neovius M. The longitudinal integrated database for health insurance and labour market studies (LISA) and its use in medical research. Eur J Epidemiol. 2019;34(4):423–37. doi: 10.1007/s10654-019-00511-8 30929112 PMC6451717

[18] Andersen JR, Hernaes UJ, Hufthammer KO, Vage V. Employment status and sick-leave following obesity surgery: a five-year prospective cohort study. PeerJ. 2015;3:e1285. doi: 10.7717/peerj.1285 26468438 PMC4592158

[19] Velcu LM, Adolphine R, Mourelo R, Cottam DR, Angus LD. Weight loss, quality of life and employment status after Roux-en-Y gastric bypass: 5-year analysis. Surg Obes Relat Dis. 2005;1(4):413–6; discussion 7. doi: 10.1016/j.soard.2005.04.007 16925260

[20] Hawkins SC, Osborne A, Finlay IG, Alagaratnam S, Edmond JR, Welbourn R. Paid work increases and state benefit claims decrease after bariatric surgery. Obes Surg. 2007;17(4):434–7. doi: 10.1007/s11695-007-9073-7 17608252

[21] Roger Andersen J, Aasprang A, Bergsholm P, Sletteskog N, Vage V, Karin Natvig G. Health-related quality of life and paid work participation after duodenal switch. Obes Surg. 2010;20(3):340–5. doi: 10.1007/s11695-009-9837-3 19352783

[22] Gripeteg L, Lindroos AK, Peltonen M, Sjostrom L, Narbro K. Effects of bariatric surgery on disability pension in Swedish obese subjects. Int J Obes (Lond). 2012;36(3):356–62. doi: 10.1038/ijo.2011.15 21364529

[23] Narbro K, Agren G, Jonsson E, Larsson B, Naslund I, Wedel H, et al. Sick leave and disability pension before and after treatment for obesity: A report from the Swedish Obese Subjects (SOS) study. Int J Obesity. 1999;23(6):619–24. 10.1038/sj.ijo.0800890 10411235

[24] Nguyen NT, Varela JE. Bariatric surgery for obesity and metabolic disorders: state of the art. Nat Rev Gastroenterol Hepatol. 2017;14(3):160–9. doi: 10.1038/nrgastro.2016.170 27899816

[25] Sjostrom L. Review of the key results from the Swedish Obese Subjects (SOS) trial—a prospective controlled intervention study of bariatric surgery. J Intern Med. 2013;273(3):219–34. doi: 10.1111/joim.12012 23163728

[26] Rantalaiho VM, Kautiainen H, Jarvenpaa S, Virta L, Pohjolainen T, Korpela M, et al. Decline in work disability caused by early rheumatoid arthritis: results from a nationwide Finnish register, 2000–8. Ann Rheum Dis. 2013;72(5):672–7. doi: 10.1136/annrheumdis-2011-200701 22679306

[27] Olofsson T, Petersson IF, Eriksson JK, Englund M, Simard JF, Nilsson JA, et al. Predictors of work disability during the first 3 years after diagnosis in a national rheumatoid arthritis inception cohort. Ann Rheum Dis. 2014;73(5):845–53. doi: 10.1136/annrheumdis-2012-202911 23520035

[28] Wamala SP, Wolk A, OrthGomer K. Determinants of obesity in relation to socioeconomic status among middle-aged Swedish women. Prev Med. 1997;26(5):734–44. doi: 10.1006/pmed.1997.0199 9327484

[29] Martin M, Beekley A, Kjorstad R, Sebesta J. Socioeconomic disparities in eligibility and access to bariatric surgery: a national population-based analysis. Surg Obes Relat Dis. 2010;6(1):8–15. doi: 10.1016/j.soard.2009.07.003 19782647

[30] Birkmeyer NJO, Gu NY. Race, Socioeconomic Status, and the Use of Bariatric Surgery in Michigan. Obesity Surgery. 2012;22(2):259–65. doi: 10.1007/s11695-010-0210-3 20559894

[31] Amick BC 3rd, Kawachi I, Coakley EH, Lerner D, Levine S, Colditz GA. Relationship of job strain and iso-strain to health status in a cohort of women in the United States. Scand J Work Environ Health. 1998;24(1):54–61. doi: 10.5271/sjweh.278 9562401

[32] Stringhini S, Carmeli C, Jokela M, Avendano M, Muennig P, Guida F, et al. Socioeconomic status and the 25 x 25 risk factors as determinants of premature mortality: a multicohort study and meta-analysis of 1.7 million men and women. Lancet. 2017;389(10075):1229–37. 28159391 10.1016/S0140-6736(16)32380-7 PMC5368415

[33] Szmulewicz A, Wanis KN, Gripper A, Angriman F, Hawel J, Elnahas A, et al. Mental health quality of life after bariatric surgery: A systematic review and meta-analysis of randomized clinical trials. Clin Obes. 2019;9(1):e12290. doi: 10.1111/cob.12290 30458582

[34] King WC, Chen JY, Mitchell JE, Kalarchian MA, Steffen KJ, Engel SG, et al. Prevalence of Alcohol Use Disorders Before and After Bariatric Surgery. Jama-J Am Med Assoc. 2012;307(23):2516–25. doi: 10.1001/jama.2012.6147 22710289 PMC3682834

[35] Svensson PA, Anveden A, Romeo S, Peltonen M, Ahlin S, Burza MA, et al. Alcohol consumption and alcohol problems after bariatric surgery in the Swedish obese subjects study. Obesity (Silver Spring). 2013;21(12):2444–51. doi: 10.1002/oby.20397 23520203

[36] Bhatti JA, Nathens AB, Thiruchelvam D, Grantcharov T, Goldstein BI, Redelmeier DA. Self-harm Emergencies After Bariatric Surgery: A Population-Based Cohort Study. JAMA Surg. 2016;151(3):226–32. doi: 10.1001/jamasurg.2015.3414 26444444

[37] Neovius M, Bruze G, Jacobson P, Sjoholm K, Johansson K, Granath F, et al. Risk of suicide and non-fatal self-harm after bariatric surgery: results from two matched cohort studies. Lancet Diabetes Endocrinol. 2018;6(3):197–207. doi: 10.1016/S2213-8587(17)30437-0 29329975 PMC5932484

