# Supplementary material for: Earnings and work loss from 5 years before to 5 years after bariatric surgery: A cohort study
Source: PLoS One. 2023 May 18;18(5):e0285379. doi: 10.1371/journal.pone.0285379 (PMC10194874; doi:10.1371/journal.pone.0285379)
Supplement: S1 Appendix — (PDF) [file pone.0285379.s001.pdf]

Supplementary Web Appendix

Earnings and work loss from 5 years before to 5 years after bariatric surgery: a cohort study

Mattias Norrbäck, PhD[1]; Martin Neovius, PhD[1]; Johan Ottosson, MD, PhD[2]; Ingmar Näslund, MD, PhD[2]; Gustaf Bruze, PhD[1]

[1]Clinical Epidemiology Division, Department of Medicine, Karolinska Institutet, Stockholm, Sweden.
[2]Department of Surgery, Faculty of Medicine and Health, Örebro University, Örebro, Sweden.

Contents

- **Supplementary Figure 1……………………………………………………………………Page 3**
- **Supplementary Figure 2……………………………………………………………………Page 4**
- **Supplementary Figure 2……………………………………………………………………Page 5**
- **Supplementary Figure 3……………………………………………………………………Page 6**
- **Supplementary Figure 4……………………………………………………………………Page 7**
- **Supplementary Figure 5……………………………………………………………………Page 8**
- **Supplementary Figure 6……………………………………………………………………Page 9**
- **Supplementary Figure 7……………………………………………………………………Page 10**
- **Supplementary Table 1……………………………………………………………………..Page 11**
- **Supplementary Table 2……………………………………………………………………..Page 12**
- **Supplementary Table 3……………………………………………………………………..Page 13**
- **Supplementary Table 4……………………………………………………………………..Page 14**

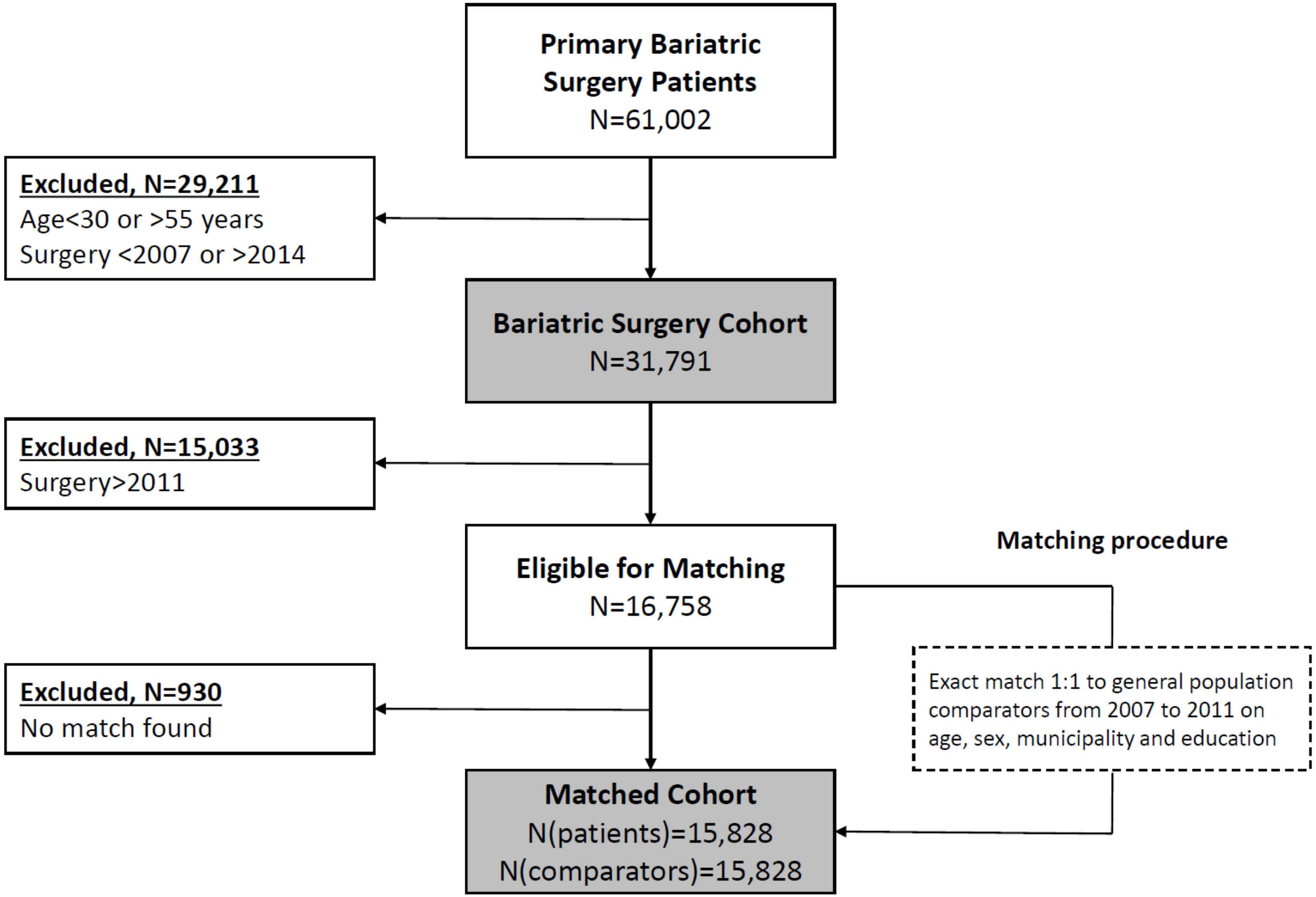

Supplementary Figure 1. Identification of the bariatric surgery cohort and the matched cohort. Bariatric patients were identified in the Scandinavian Obesity Surgery Registry (SOReg) and comparators were identified in the Swedish Total Population Register. A population comparator had to be alive at the time of surgery to be eligible for matching.

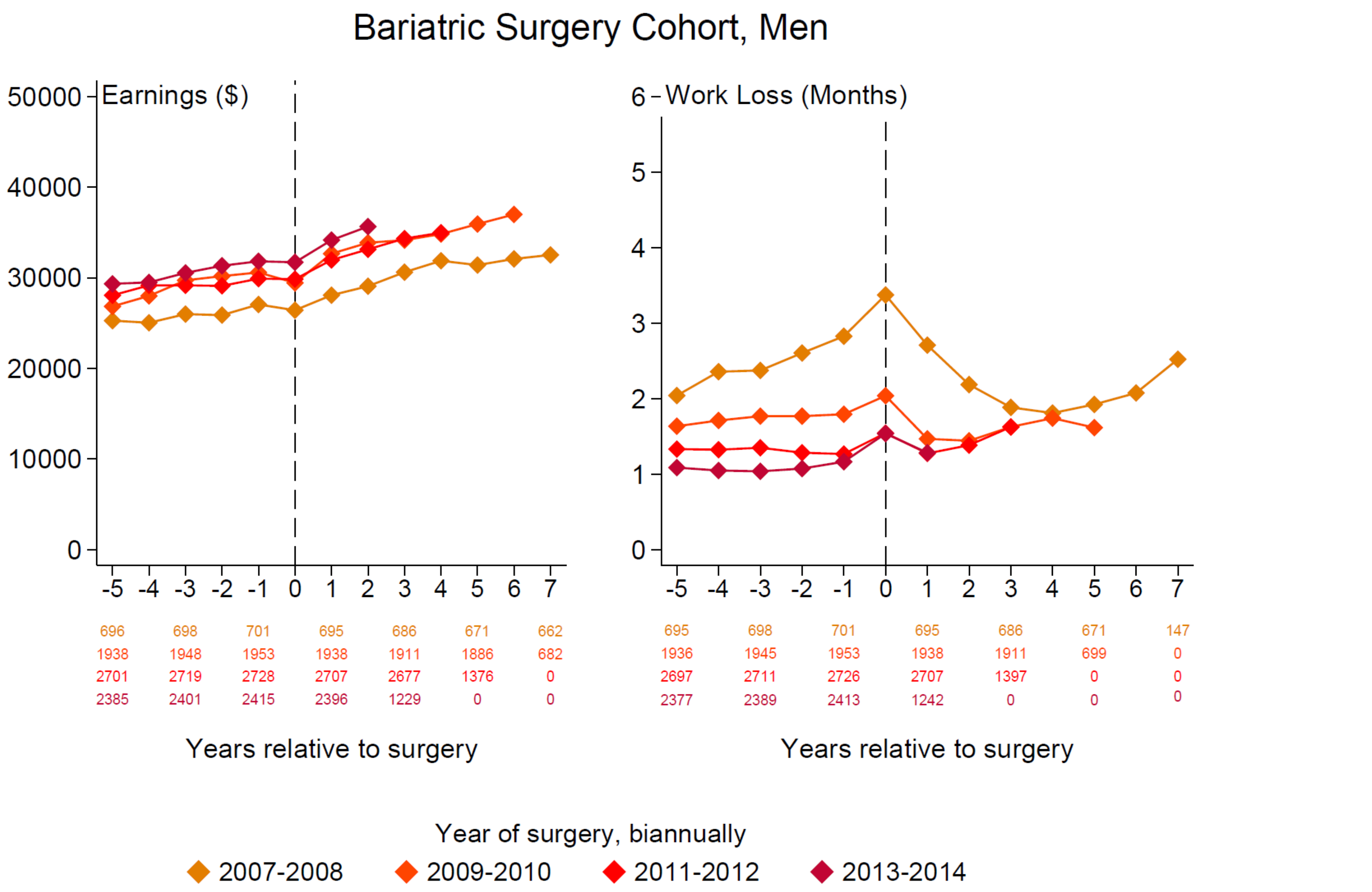

Supplementary Figure 2. Development of taxable annual earnings in 2016 U.S. dollars and work loss for the bariatric surgery patients, by sex (men upper panel, women lower panel). Vertical dashed line is surgery year.

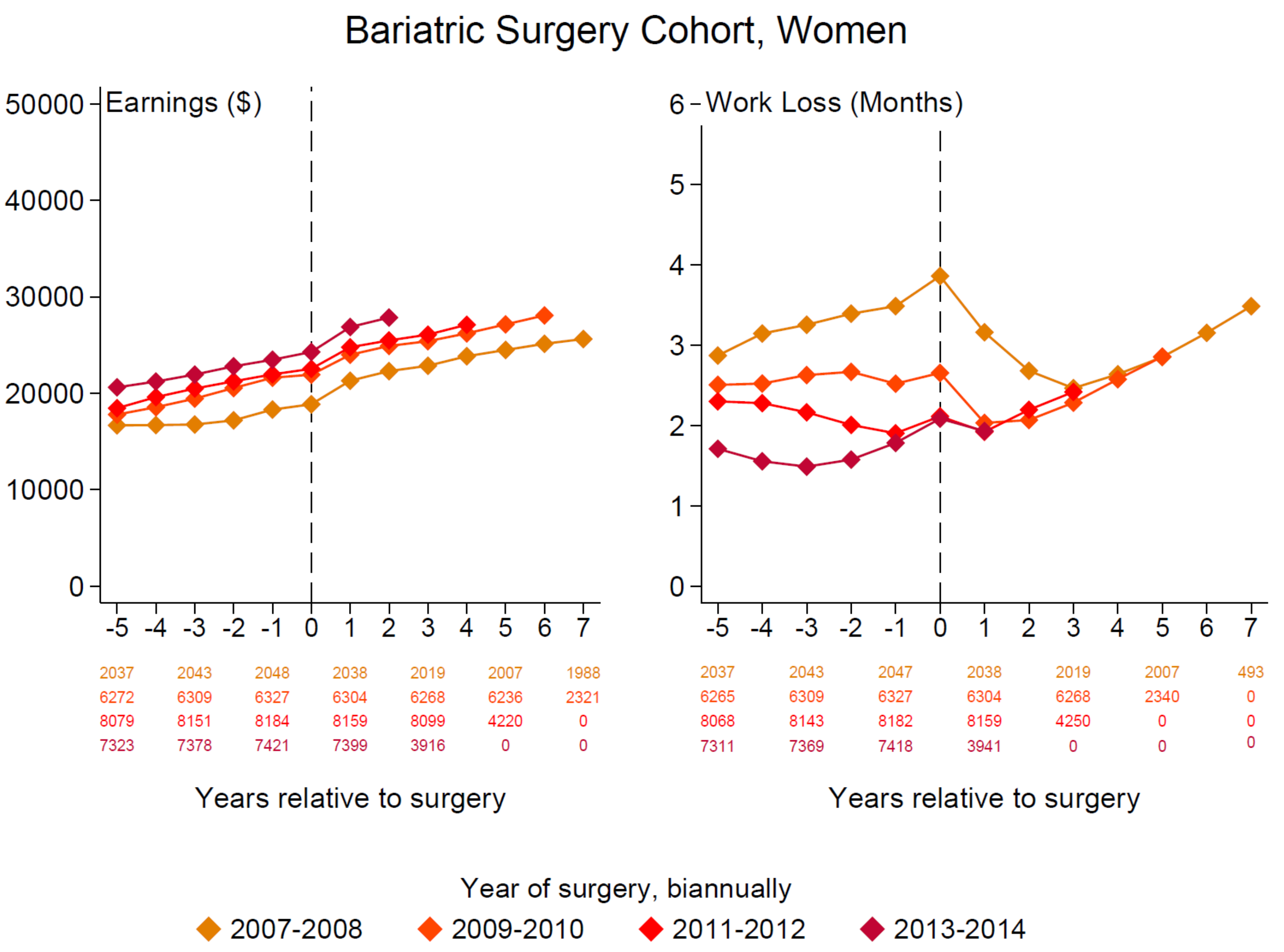

Supplementary Figure 2. Development of taxable annual earnings in 2016 U.S. dollars and work loss for the bariatric surgery patients, by sex (men upper panel, women lower panel). Vertical dashed line is surgery year.

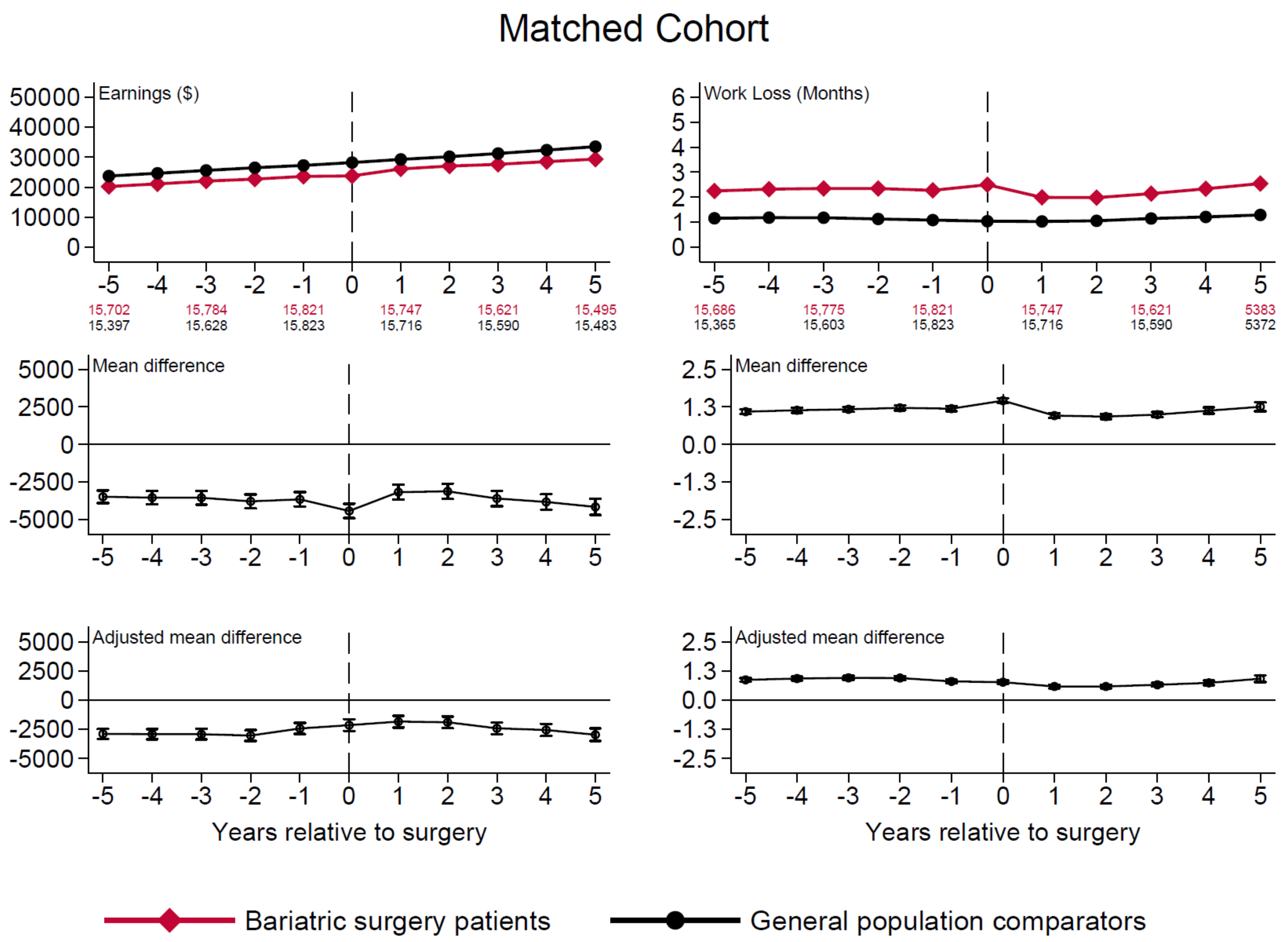

Supplementary Figure 3. Development of taxable annual earnings in 2016 U.S. dollars and work loss for the bariatric surgery patients and comparators from the general population matched on age, sex, place of residence, and educational level (primary school, high school, or university training). Calculated mean differences are earnings (or work loss) for bariatric surgery patients minus earnings (or work loss) for comparators from the general population. Mean differences were further adjusted for marital status, annual number of hospital days, and annual outpatient hospital visits (bottom right and left panels). Vertical dashed line is surgery year for patients and index year for general population comparators.

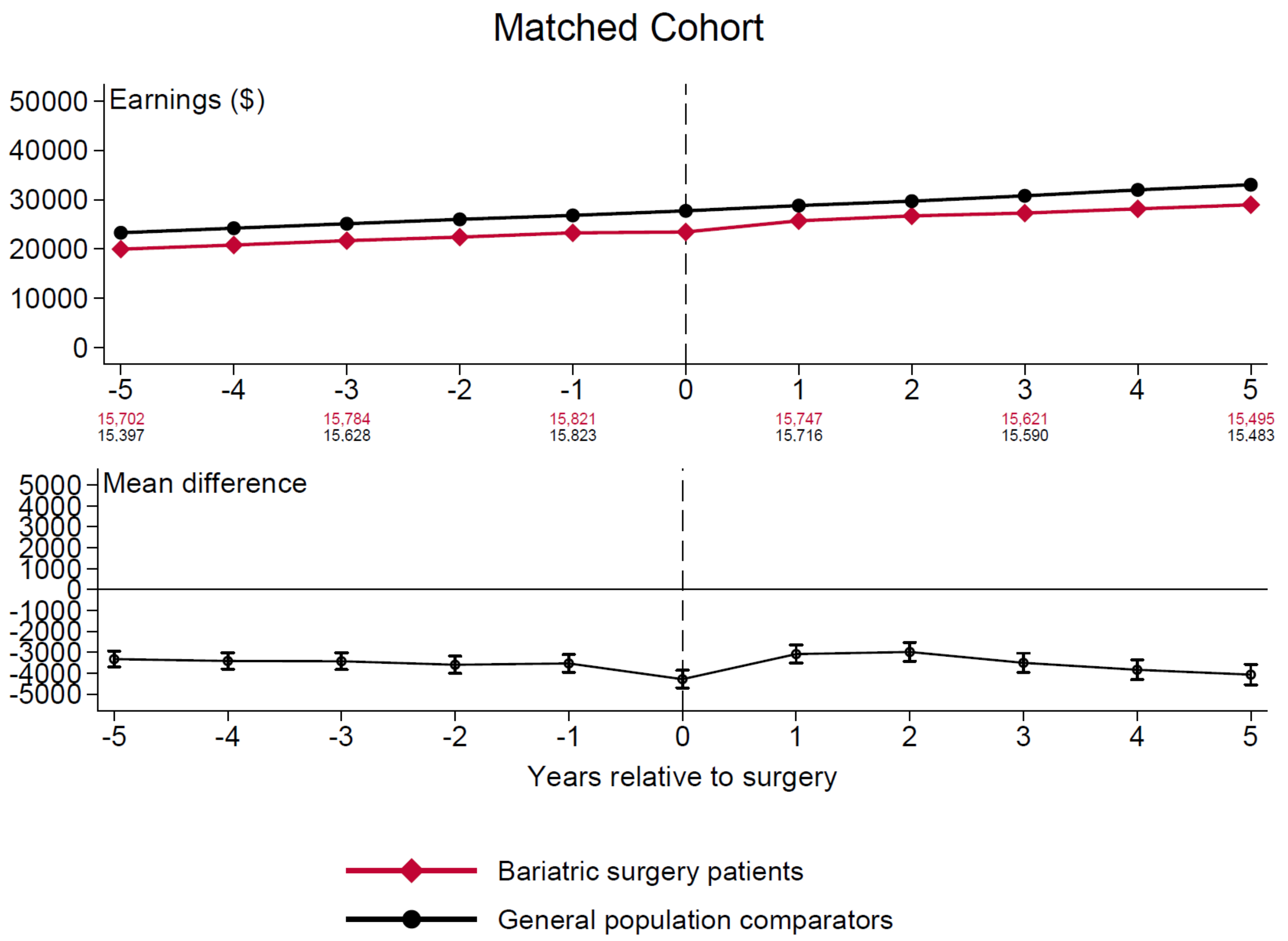

Supplementary Figure 4. Development of taxable annual earnings in 2016 U.S. dollars for the bariatric surgery patients and comparators from the general population matched on age, sex, place of residence, and educational level (primary school, high school, or university training). Calculated mean differences are earnings for bariatric surgery patients minus earnings for comparators from the general population. Mean differences were calculated after winsorizing annual taxable earnings at the 1st and 99th percentile. Vertical dashed line is surgery year for patients and index year for general population comparators.

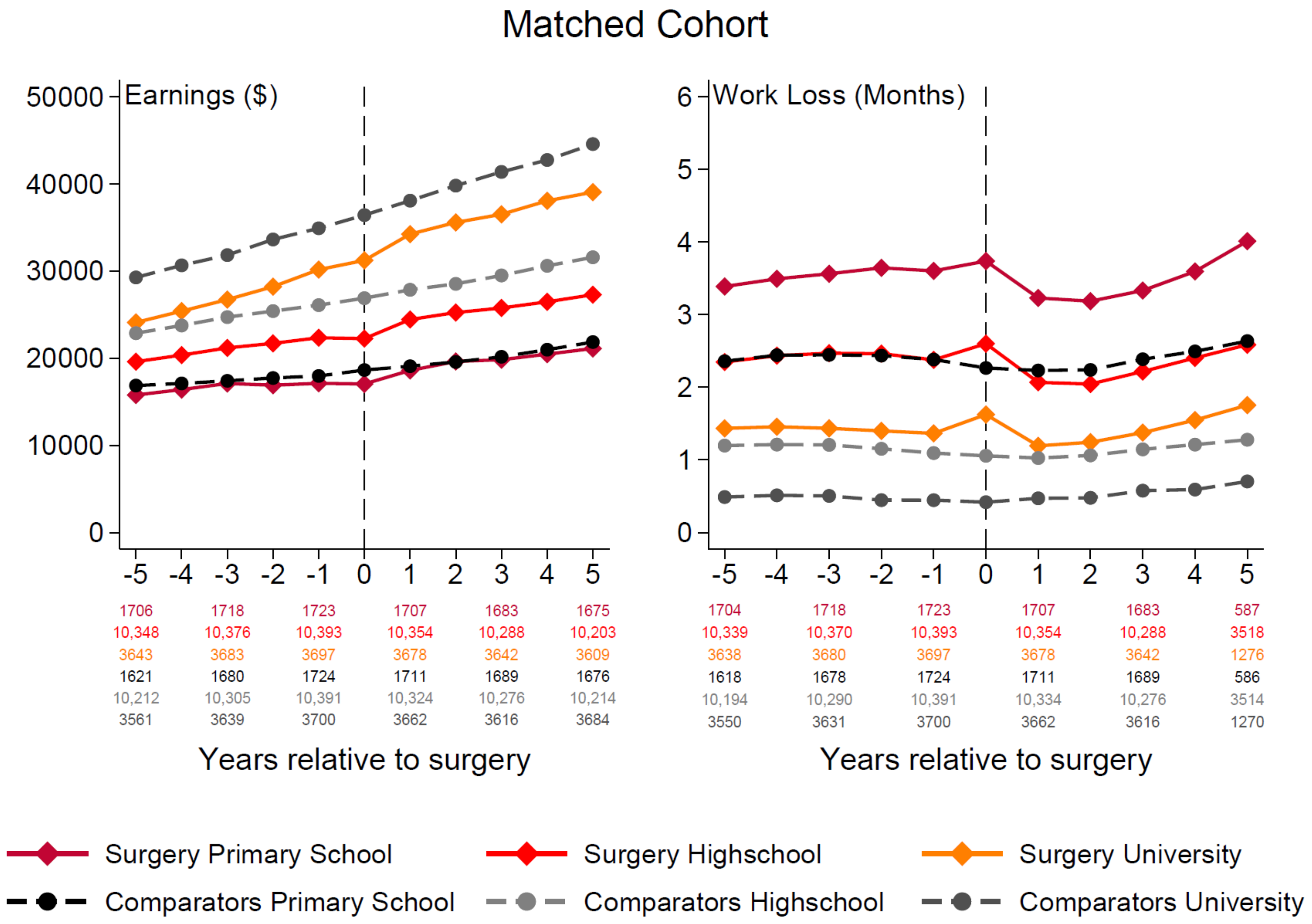

Supplementary Figure 5. Development of annual earnings in 2016 U.S. dollars and work loss for bariatric surgery patients and comparators from the general population matched on age, sex, place of residence, and educational level (primary school, high school, or university training), by educational level. Vertical dashed line is surgery year for patients and index year for general population comparators.

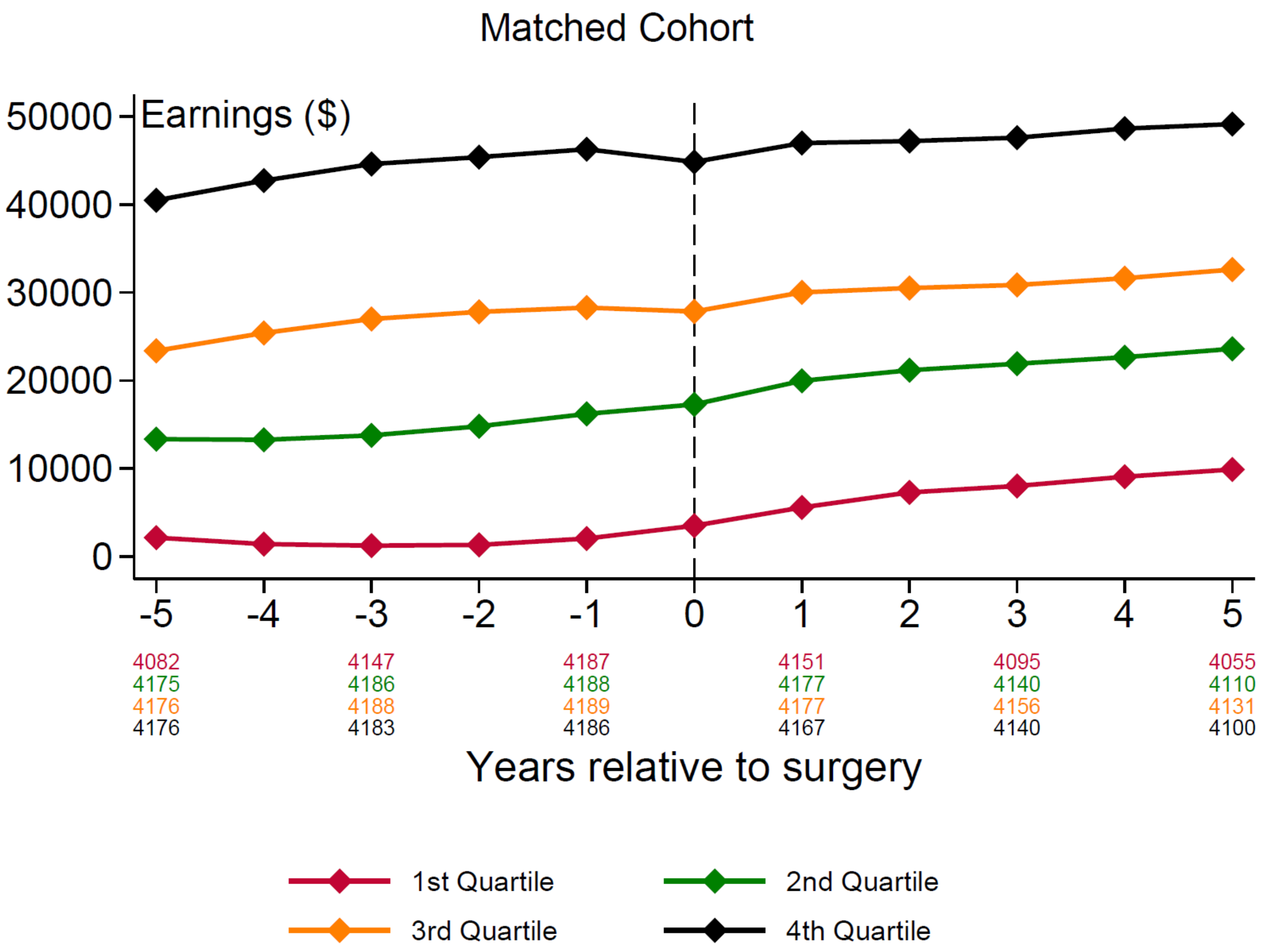

Supplementary Figure 6. Development of taxable annual earnings in 2016 U.S. dollars for the bariatric surgery patients and comparators from the general population matched on age, sex, place of residence, and educational level (primary school, high school, or university training), by earnings quartiles before surgery. Vertical dashed line is surgery year for patients and index year for general population comparators.

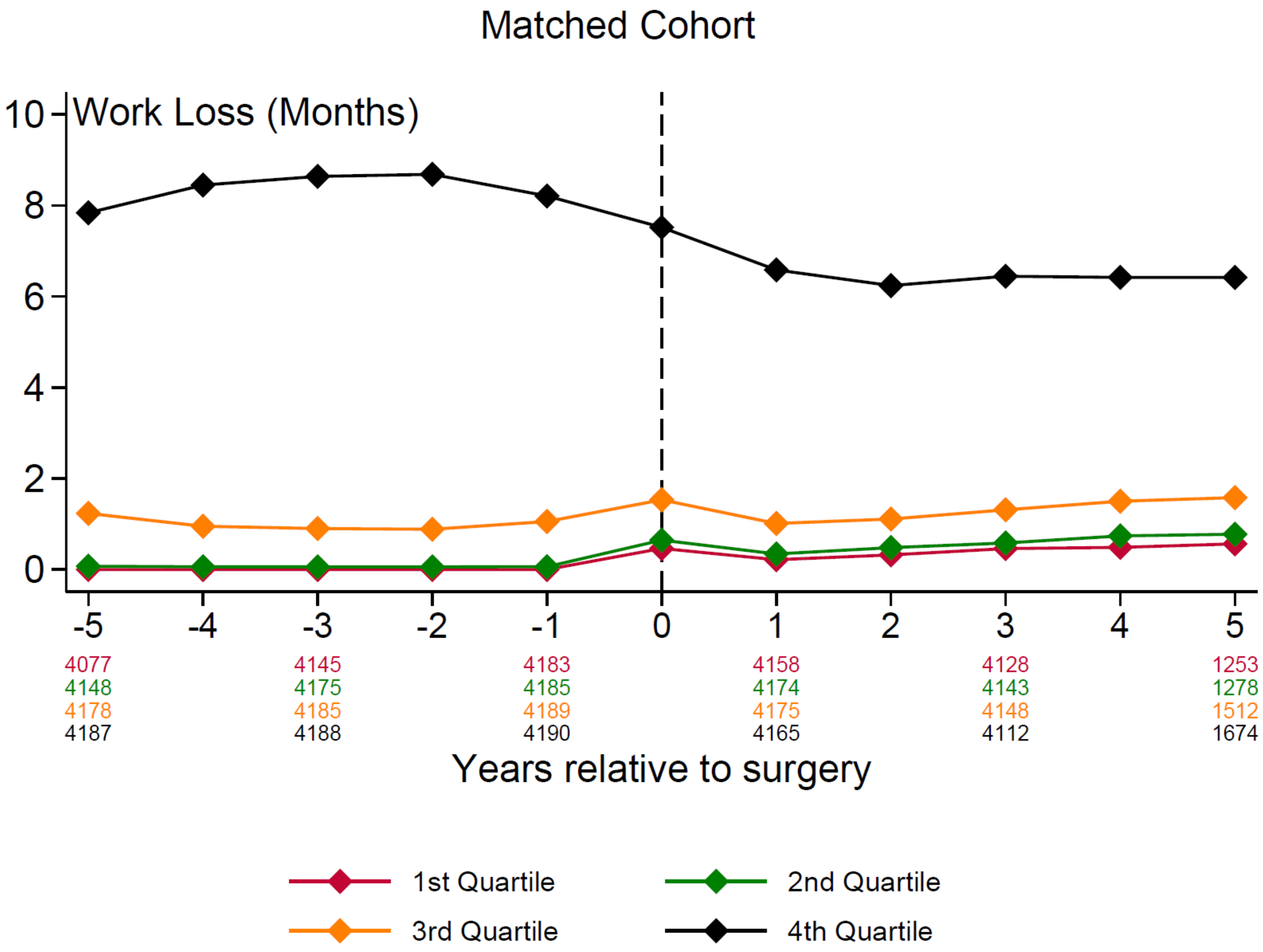

Supplementary Figure 7. Development of work loss for the bariatric surgery patients and comparators from the general population matched on age, sex, place of residence, and educational level (primary school, high school, or university training), by earnings quartiles before surgery. Vertical dashed line is surgery year for patients and index year for general population comparators.

Supplementary Table 1. International Classification of Diseases (ICD) codes and Anatomical Therapeutical Category (ATC) codes used.

	Register	ICD codes	ATC	Comment
Circulatory disease	National Patient Register	ICD-8: 390–458<br>ICD-9: 390–459<br>ICD-10: Chapter I		ICD codes from inpatient and outpatient care
Psychiatric disease	National Patient Register	ICD-8: 290–315<br>ICD-9: 290–319<br>ICD-10: Chapter F		ICD codes from inpatient and outpatient care
Lipid lowering therapy	Swedish Prescribed Drug Register (July 2005-)		C10AA-C10AD, C10AX, C10B	
Antidiabetic drug use	Swedish Prescribed Drug Register (July 2005-)		A10	Oral diabetes drugs, insulins
Antihypertensive therapy	Swedish Prescribed Drug Register (July 2005-)		C02<br>C03A, C03EA01<br>C07 (excl. C07AA07)<br>C08C<br>C09A, C09B<br>C09C, C09D<br>C09X	ARB<br>Thiazide diuretics<br>Beta blockers<br>Calcium antagonists, vessel selective<br>ACE-inhibitors<br>Alfa blockers and other centrally acting drugs<br>Renin inhibitors
Psychotropic drug use	Swedish Prescribed Drug Register (July 2005-)		N05, N06	N05A: Antipsychotics<br>N05B: Anxiolytics<br>N05C: Hypnotics and sedatives<br><br>N06A: Antidepressants<br>N06B: Psychostimulants<br>N06C: Psycholeptics and psychoanaleptics<br>N06D: Anti-dementia drugs

Supplementary Table 2. Characteristics of the bariatric surgery patients.

	Year of surgery	
**Bariatric surgery patients**	**2007-2014**	**2007-2011†**
N	31,791	16,758
Women, n (%)	23,989 (75.5)	12,674 (75.6)
Age (years)	42.2 (6.9)	41.9 (6.8)
Pre-surgery BMI (kg/m^2)	42.2 (5.7)	42.9 (5.7)
**Year of surgery, n (%)**		
2007-2008	2752 (8.7)	2752 (16.4)
2009-2010	8283 (26.1)	8283 (49.4)
2011-2012	10,915 (34.3)	5723 (34.2)
2013-2014	9841 (31.0)	-
**Procedure type, n (%)**		
Gastric bypass	29,952 ()	16,351 (97.6)
Sleeve	1364 (4.3)	78 (0.5)
Other	475 (1.5)	329 (2.0)
**Educational level, n (%)**		
Primary school	4836 (15.2)	2506 (15.0)
High school	19,393 (61.0)	10,444 (62.3)
University	7438 (23.4)	3758 (22.4)
Education missing	124 (0.4)	50 (0.3)
**Income ($*)**		
Mean (SD)	24,100 (20,600)	23,200 (21,000)
25th percentile	3400	2200
50th percentile(median)	25,600	24,300
75th percentile	36,400	35,500
**Work loss months, Mean (SD)**	2.0 (4.0)	2.3 (4.2)
Sick leave	0.8 (2.4)	0.8 (2.4)
Disability pension	1.2 (3.3)	1.5 (3.7)

BMI = Body Mass Index

* Collected the year before surgery. Currency units presented as 2016 USD (converted from Swedish crowns, based on exchange rate July 1st, 2016, adjusted for Swedish annual inflation according to Swedish CPI)

† A subset of bariatric surgery patients that are matched to population comparators ensuring follow-up from 5y before to 5y after the year of surgery

Supplementary Table 3. Annual earnings among bariatric surgery patients and matched comparators from the general population from 5 years before to 5 years after surgery.

	**Earnings and 95% confidence intervals**	
Years relative to surgery	Bariatric surgery patients	Matched comparators from the general population
-5	20200 (19900 – 20500)	23700 (23400 – 24000)
-4	21100 (20800 – 21400)	24600 (24300 – 25000)
-3	22000 (21700 – 22300)	25600 (25200 – 25900)
-2	22700 (22400 – 23000)	26500 (26100 – 26800)
-1	23600 (23300 – 23900)	27300 (26900 – 27600)
0	23800 (23400 – 24100)	28200 (27800 – 28600)
1	26100 (25700 – 26400)	29300 (28900 – 29600)
2	27000 (26700 – 27400)	30200 (29800 – 30500)
3	27600 (27300 – 28000)	31200 (30900 – 31600)
4	28500 (28100 – 28900)	32400 (32000 – 32700)
5	29300 (29000 – 29700)	33500 (33100 – 33900)

Values are 2016 US dollars using the exchange rate from July 1st 2016 (1 $USD = 8.43 SEK)

Supplementary Table 4. Annual work loss among bariatric surgery patients and matched comparators from the general population from 5 years before to 5 years after surgery.

	**Work loss and 95% confidence intervals**	
Years relative to surgery	Bariatric surgery patients	Matched comparators from the general population
-5	2.25 (2.18 – 2.31)	1.16 (1.11 – 1.21)
-4	2.32 (2.25 – 2.39)	1.18 (1.13 – 1.23)
-3	2.35 (2.28 – 2.41)	1.18 (1.13 – 1.23)
-2	2.34 (2.28 – 2.41)	1.13 (1.08 – 1.18)
-1	2.27 (2.21 –2.34)	1.08 (1.03 – 1.13)
0	2.50 (2.43 – 2.56)	1.04 (0.99 – 1.09)
1	1.99 (1.93 – 2.05)	1.03 (0.98 –1.07)
2	1.98 (1.92 – 2.04)	1.05 (1.01 – 1.10)
3	2.14 (2.08 – 2.20)	1.15 (1.10 –1.20)
4	2.33 (2.25 – 2.42)	1.21 (1.15 – 1.27)
5	2.54 (2.43 – 2.66)	1.29 (1.20 – 1.38)

Values are combined days of sick leave and disability pension displayed as total months in a year.
